# Age-Related Disease Association of Endogenous γ-H2AX Foci in Mononuclear Cells Derived from Leukapheresis

**DOI:** 10.1371/journal.pone.0045728

**Published:** 2012-09-21

**Authors:** Shepherd H. Schurman, Christopher A. Dunn, Rebecca Greaves, Binbing Yu, Luigi Ferrucci, Deborah L. Croteau, Michael M. Seidman, Vilhelm A. Bohr

**Affiliations:** 1 Laboratory of Molecular Gerontology, National Institute on Aging, National Institutes of Health, Baltimore, Maryland, United States of America; 2 Laboratory of Epidemiology, Demography and Biometry, National Institute on Aging, National Institutes of Health, Baltimore, Maryland, United States of America; 3 Clinical Research Branch, National Institute on Aging, National Institutes of Health, Baltimore, Maryland, United States of America; University of Louisville, United States of America

## Abstract

The phosphorylated form of histone H2AX (γ-H2AX) forms immunohistochemically detectable foci at DNA double strand breaks. In peripheral blood mononuclear cells (PBMCs) derived from leukapheresis from patients enrolled in the Baltimore Longitudinal Study of Aging, γ-H2AX foci increased in a linear fashion with regards to age, peaking at ∼57 years. The relationship between the frequency of γ-H2AX foci and age-related pathologies was assessed. We found a statistically significant (*p* = 0.023) 50% increase in foci in PBMCs derived from patients with a known history of vitamin D deficiency. In addition, there were trends toward increased γ-H2AX foci in patients with cataracts (34% increase, *p*<0.10) and in sleep apnea patients (44%, *p*<0.10). Among patients ≥57 y/o, we found a significant (p = 0.037) 36% increase in the number of γ-H2AX foci/cell for patients with hypertension compared to non-hypertensive patients. Our results support a role for increased DNA damage in the morbidity of age-related diseases. γ -H2AX may be a biomarker for human morbidity in age-related diseases.

## Introduction

Preserving genome integrity and stability are crucial for cellular function. Loss of integrity through DNA damage, which increases progressively with age, may contribute to diseases related to aging [1]. DNA double-strand breaks (DSBs), formed when both DNA strands are broken in close proximity (<20 bp), can severely damage genome integrity and threaten cell survival [2]. Cells have developed protective responses to DNA damage, including lesion repair, damage tolerance, and checkpoint pathways [1]. The inability to properly repair DSBs has been linked to several genetic diseases including, ataxia telangiectasia, ataxia telangiectasia-like syndrome, Nijmegen breakage syndrome, Fanconia anemia, and individuals with heterozygous *BRCA1* or *BRCA2* mutations who exhibit a greatly increased risk of breast and ovarian cancer [3]. There is an increased susceptibility to cancer in many of these syndromes.

Following DSBs formation, histone H2AX is phosphorylated on serine residue 139, thereby generating γ-H2AX, which serves as a platform to recruit additional factors [4]. The phosphorylation of H2AX is readily detected by immunofluorescence and provides a good marker for the earliest and most robust post-translational histone modification following a DSB [5]. Within seconds of a DSB, γ-H2AX spreads over a region spanning between thousands and millions of DNA bases [4], [5], perhaps encompassing regions of up to 30 Mbps [6], [7]. Though it is believed that each DSB corresponds to one γ-H2AX focus, the reverse is not true as γ-H2AX may persist for hours after DSBs are rejoined [8], [9]. During the early stages of the DNA damage response, a high number of small foci are formed which decrease in number and increase in size as the DNA damage response progresses [8].

The finding by Sedelnikova *et al.* [10] of cryptogenic γ-H2AX foci in senescing human cells in culture and in aging mice suggested that there were un-repairable DSBs that accumulate with age. A follow-up study [11] examining human peripheral blood lymphocytes found that the incidence of endogenous γ-H2AX foci increased with age until ∼50 years old and were considerably higher in age-matched Werner syndrome patients, a disorder characterized by segmental premature aging, genomic instability and a phenotype that resembles accelerated aging. The study was limited by the small number of participants (eight) over the age of 50, and an age range to 72.

These results prompted us to analyze DSBs in samples from individuals with diseases commonly associated with aging. Given the ready availability of blood samples, lymphocytes have been used as a surrogate when other tissues are not available. Many studies have used conventional methods to measure DSBs including the Comet assay or constant field gel electrophoresis/pulse field gel electrophoresis. They have several disadvantages compared with γ-H2AX detection including the need for 5–50 Gy of ionizing radiation to detect DSBs [12], and the need for lysis at high temperatures with the Comet assay [8]. The detection of γ-H2AX is more sensitive than other methods used to detect DSBs at clinically relevant doses of genotoxic stressors [8].

The Baltimore Longitudinal Study of Aging (BLSA) at the National Institute on Aging is the longest running longitudinal study of aging in America. This on-going population-based cohort study of age–related diseases allows detailed follow-up of patients’ conditions and monitoring of a large number of clinical parameters. Many BLSA participants undergo a 2-hour leukapheresis, which provides peripheral blood cells (PBMCs, 0.5–1.0×10^10^ cells) for many researchers to perform simultaneous experiments. We have used leukapheresis-derived PBMCs from BLSA participants to study the relationship between γ-H2AX foci and age in patients over-50, N = 37. We have also tested the significance of correlations between γ-H2AX foci and exposure to environmental stressors such as irradiation treatment for prostate cancer, chronic hypoxia (as found in sleep apnea) and vitamin D deficiency. In addition, we have explored whether BLSA participants affected by diseases with a strong genetic component such as cancer, cataracts and hypertension have different numbers of γ-H2AX foci compared to participants not affected by these diseases. To our knowledge, this is the first study that correlates H2AX phosphorylation with age-related disorders, in a single cohort.

## Results

**Figure 1:**
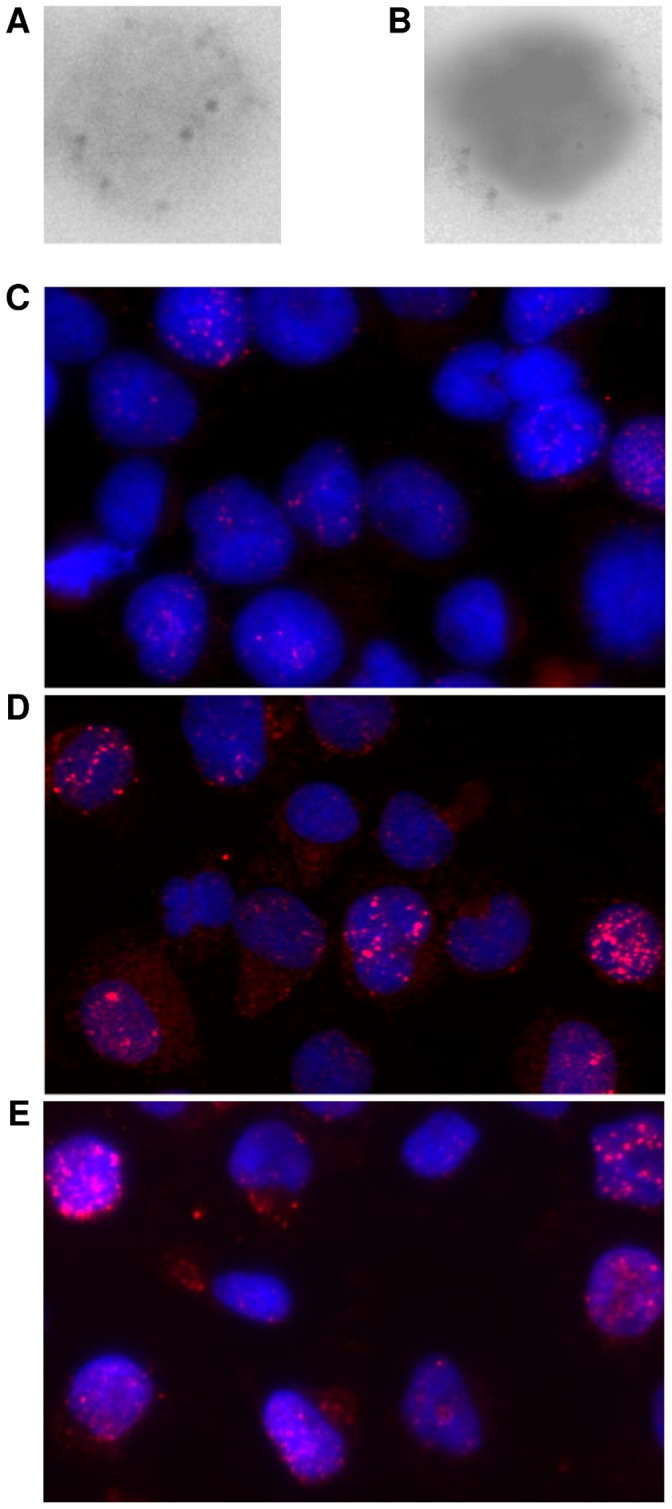
γ-H2AX foci in human leukapheresis-derived mononuclear cells and transformed lymphocytes. (**A**) Human leukapheresis-derived mononuclear cells, stained for γ-H2AX foci. Image post preset automated algorithm for image analysis using Adobe Photoshop, version 7.0. Preset automated algorithm for image analysis consists of: ‘auto contrast,’ ‘auto levels,’ ‘desaturate,’ ‘invert.’ Without DAPI overlay, γ-H2AX foci which fell within the area of the nucleus were easier to identify and count. (**B**) Human leukapheresis-derived mononuclear cells, stained for γ-H2AX foci and overlaid with DAPI stained nuclear DNA antibody. Image post preset as described in Figure 1A. With DAPI overlay, γ-H2AX foci were more difficult to identify and count. (**C–E**) γ-H2AX foci in human transformed lymphocytes, Cytocentrifugation at various speeds before fixation. C. 200 rpm, D. 350 rpm, E. 500 rpm. Severity of DNA damage increases with higher G force settings.

γ-H2AX foci within nuclei were quantified as per experimental procedures (**Figs 1A and 1B**). While our study of leukapheresis derived samples used fixed cells, we also examined live unfixed cells (transformed human lymphocytes) for the effects of cytocentrifugation at higher speeds (200, 350, and 500 rpm with correspondingly greater G force). We found that using higher centrifugation speed in these unfixed cells resulted in an increase of γ-H2AX foci (**Figs 1C, 1D and 1E**).

In our leukapheresis derived samples from BLSA participants using fixed cells, we found an average of 4.25 γ-H2AX foci/cell. The average number of γ-H2AX foci/cell for men vs. women was 4.53±0.35 and 3.75±0.45 (*p* = 0.25).

**Figure 2:**
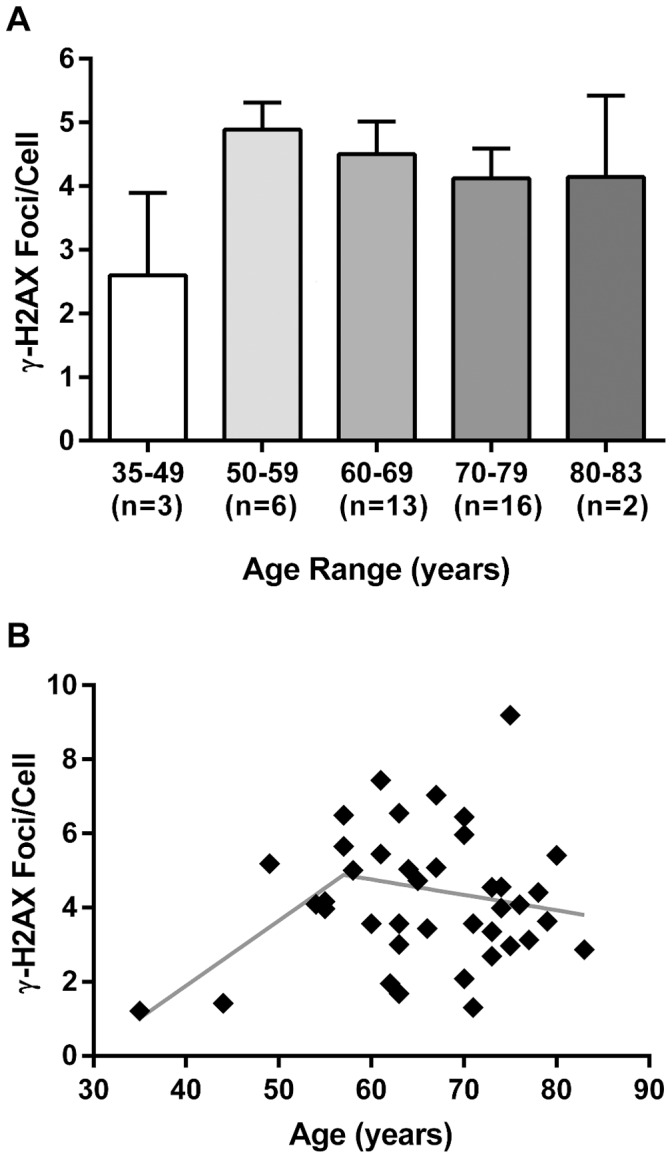
Distribution of γ-H2AX foci/cell in human leukapheresis-derived mononuclear cells by age. (**A**) γ-H2AX foci in human leukapheresis-derived mononuclear cells, stratified by age subgroups. Average γ-H2AX foci/cell is shown for each age subgroup. (**B**) Scatter plot of γ-H2AX foci/cell in human leukapheresis-derived mononuclear cells by age. Dotted line is best fit showing change-point (age 57) as determined by piecewise linear regression.

**Figure 2A** shows the distribution γ-H2AX foci/cell averages for all patients by age decade. γ-H2AX foci/cell averages were 4.89, 4.53, 4.12 and 4.14 for the 50–59, 60–69, 70–79 and 80–83 year age groups, respectively. Using piecewise linear regression analysis [13], γ-H2AX foci/cell in our leukapheresis derived samples from BLSA participants had a change-point at age 57. By adding a change point at 57, the R-square statistic increased from 0.015 to 0.142. Therefore, the change-point model yielded a much better fit than the simple linear regression model. The final fitted model with a change point at age 57 is expressed by the regression equation



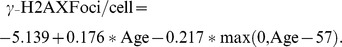



The corresponding slopes for the two segments are 0.176 (0.176–0 in the equation) and −0.041 (0.176–0.217 in the equation). The standard errors, 95% confidence intervals and p-values of the two slopes are (0.071, 0.094–0.258, 0.018) for the first slope and (0.032, −0.105–0.021, 0.191) for the second. This shows a significant increase in γ-H2AX levels to the change-point at 57 years old followed by a slightly decreasing but non-significant trend of γ-H2AX foci/cell with age thereafter (**Figure 2B**, illustrated as a scatter plot). The difference of the slopes between the two segments is −0.217 (0.093 standard error, *p* = 0.025), which shows the slopes change significantly before and after the change-point at age 57.

**Figure 3:**
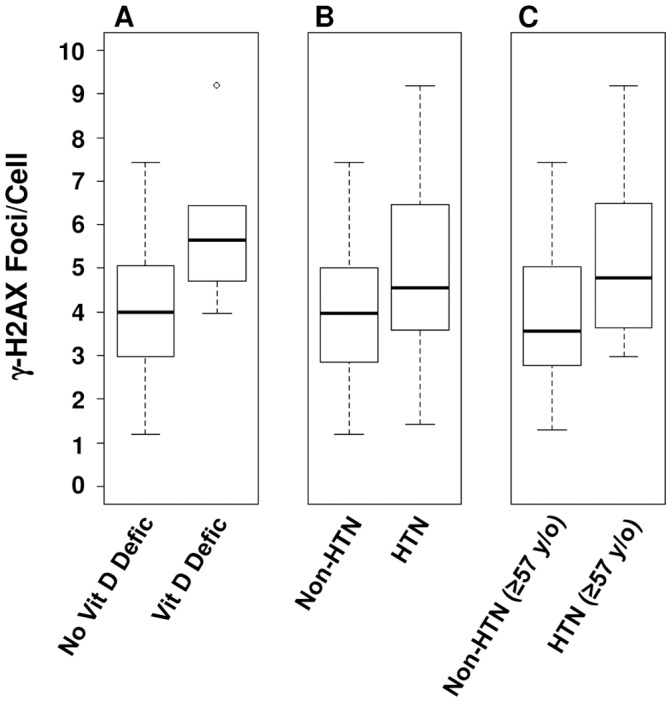
Box plots of γ-H2AX foci/cell in mononuclear cells by age for patients with vitamin D deficiency and hypertension. (**A**) Box plot of γ-H2AX foci/cell in human leukapheresis-derived mononuclear cells by age for patients with vitamin deficiency compared to those with no known vitamin D deficiency. (**B**) Box plot of γ-H2AX foci/cell in human leukapheresis-derived mononuclear cells by age for hypertensive patients vs. non-hypertensive patients. (**C**) Box plot of γ-H2AX foci/cell in human leukapheresis-derived mononuclear cells by age for hypertensive patients vs. non-hypertensive patients, age ≥57 y/o. The top of each box in the box plots indicates the 75^th^ percentile, the bottom of each box indicates the 25^th^ percentile and the bar inside the box is the median. The whiskers extend out to the most extreme data point that is at most 1.5 times the interquartile range above the third quartile or below the first quartile. The circle in Figure 3 (A) indicates a result from a patient above this range.

The number of γ-H2AX foci/cell among BLSA participants with positive history or evidence of cancer irradiation treatment [14], sleep apnea (reduced oxygenation) [15], [16] and vitamin D deficiency[17]–[19], compared to those without history of these conditions is shown in **Table 1**. Patients with previous cancer irradiation (therapy) or sleep apnea had an increased number of H2AX foci/cell (6.00±0.42 and 5.92±1.64, respectively) compared with those not known to have these conditions (4.11±0.27 and 4.15±0.32, respectively) but did not reach statistical significance (*p* = 0.23 and *p* = 0.097, respectively). Patients with known vitamin D deficiency had a significant (*p* = 0.023) 50% increase in the average number of γ-H2AX foci/cell compared with those not known to have vitamin D deficiency (5.99±0.90 vs. 4.00±0.27) (**Fig. 3A** is a box plot depicting those with vitamin D deficiency compared to those with no known vitamin D deficiency). As expected, a diagnosis of benign prostatic hyperplasia had no effect on the number of γ-H2AX foci/cell compared to the entire study group.

**Table 1:** γ-H2AX foci/cell in human leukapheresis-derived mononuclear cells, stratified by disease subgroups which contribute to oxidative stress and/or DNA damage (with the exception of benign prostatic hyperplasia).

Sub Group (n)	γ-H2AX Foci/Cell	SEM	% Increase of γ-H2AX Foci/Cell from sub-group without known condition (% decrease)	Significance (adjusted for age and sex)
All patients (40)	4.25	±0.28		
Men (26)	4.53	±0.35		
Women (14)	3.75	±0.45		
Men vs. Women				*p* = 0.25#
**Disease Subgroups**				
Cancer Irradiation Treatment (2)	6.00	±1.44	44%	*p* = 0.23
Sleep Apnea (3)	5.92	±1.64	44%	*p* = 0.097
Vitamin D Deficient (5)	5.99	±0.90	50%	*p* = 0.023
Benign Prostatic Hyperplasia (7)	3.74	±0.50	(14%)	*p* = 0.17
Any cancer (8)*	4.23	±0.65	(2%)	*p* = 0.88
Visceral cancer (4)	5.03	±0.89	21%	*p* = 0.42
Basal cell cancer (5)	3.74	±0.74	(15%)	*p* = 0.55
Cataract Formation (4)	5.51	±1.30	34%	*p* = 0.099

Average γ-H2AX foci/cell is shown for each disease subgroup and significance is adjusted for age and sex. Vitamin D deficient patients show an increase of 41% of γ-H2AX foci/cell compared to the cells from those without known vitamin D deficiency (*p* = 0.023). There is also a similar strong trend (30–39% increase in average number of γ-H2AX foci/cell, that does not quite reach significance; *p* values <0.10) in cells derived from patients with cataract formation and sleep apnea. Patients with cancer irradiation treatment show an increase in H2AX foci/cell (41%) but did not reach statistical significance (*p* = 0.23). Patients with cancer (Basal cell carcinoma or visceral cancer which includes prostate and colon cancer) showed no significant differences. Patients with benign prostatic hyperplasia also did not show any significant differences, as expected.

* one patient had both a visceral cancer and a basal cell carcinoma.

# adjusted for age.

**Figure 4:**
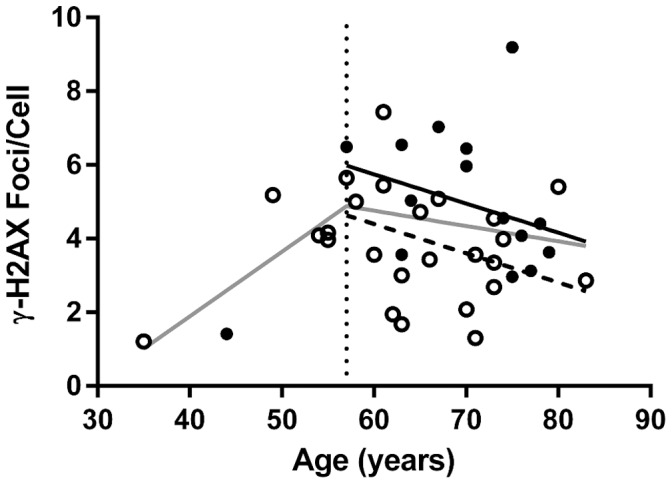
Scatterplot of γ-H2AX foci/cell in mononuclear cells by age for patients with and without hypertension. Solid circles indicate patients with hypertension; hollow circles indicate those without hypertension. Solid line indicates best fit line for hypertension patients age 57 and over. Dashed line indicates best fit line for non-hypertensive patients age 57 and over. Light line indicates regression curve for all patients.

Visceral cancers (prostate and colon) and skin cancers (basal cell carcinoma) or a combination of the two were not associated with a differential number of γ-H2AX foci/cell compared to the whole group. Participants with cataracts had an increase in γ-H2AX foci/cell compared to those without cataracts (5.51±1.30 compared to 4.11±0.28), but the difference was not significant (*p* = 0.099). Though hypertensive participants had a statistically significant (*p* = 0.046) 30% increase in γ-H2AX foci/cell when compared to non-hypertensive participants (4.97±0.51 vs. 3.82±0.30), this significance did not hold up when adjusted for age and sex (*p* = 0.13) (**Table 2**). However, when the analysis was limited to the ≥57 year old subgroup where the effect of age on γ-H2AX foci/cell had stabilized, analysis revealed a statistically significant (*p* = 0.037) 36% increase in γ-H2AX foci/cell when compared to non-hypertensive participants (5.22±0.36 vs. 3.84±0.32) even after adjusting for age and sex. **Figure 3B** is a box plot depicting those with hypertension compared to those without hypertension. **Figure 3C** is a box plot depicting those with hypertension compared to those without hypertension, limited to the ≥57 year old subgroup. Interestingly, of the seven individuals (six men and one women) with the highest γ-H2AX foci/cell (5.97 or greater, 40% or more above average), all but one were hypertensive (one man was non-hypertensive), see scatter plot (**Figure 4**) of hypertensive vs non-hypertensive patients for comparison.

**Table 2:** γ-H2AX foci/cell in human leukapheresis-derived mononuclear cells for hypertensive and non-hypertensive patients.

Sub Group (n)	γ-H2AX Foci/Cell	SEM	% Increase of γ-H2AX Foci/Cell from sub-group without known condition	Significance
**All Ages**				
Hypertension (15)	4.97	±0.51		
Non-hypertension (25)	3.81	±0.30		
Hypertensive vs. Non-hypertensive, unadjusted			30%	*p* = 0.046
Hypertensive vs. Non-hypertensive, adjusted for age and sex			30%	*p* = 0.13
**Age ≥57**				
Hypertension (14)	5.22	±0.36		
Non-hypertension (20)	3.84	±0.32		
Hypertensive vs. Non-hypertensive, adjusted for age and sex			36%	*p* = 0.037

Mononuclear cells from patients with hypertension age 57 and older show a significant (*p* = 0.037, adjusted for age and sex) increase of 30% of γ-H2AX foci/cell compared to the cells from non-hypertensive patients in the same age group.

## Discussion

Studies of DNA damage in cells have provided important clues and hypotheses on the pathophysiology of human disease. Here we have used data from the BLSA to explore how the number of γ-H2AX foci correlates with age and chronic conditions that have been associated with the accumulation of genome damage.

Sedelnikova *et al.* [11] reported an increase in γ-H2AX levels in younger adults (through age 50) and then a leveling off thereafter. Their over-50 group contained eight patients and the oldest was age 72. A major strength of the BLSA is the preponderance of patients over age 50 and one of our major goals was to more closely examine this age group with a larger cohort of patients and extend the age of the oldest patients examined. Using the BLSA series, we were able to analyze 37 patients over the age of 50, through the age of 83 (there were 13 patients over the age of 72). Using piecewise linear regression, we detected a rise in γ-H2AX levels to a change-point at 57 years old with a slight downward slope of γ-H2AX foci/cell with age thereafter (**Figure 2B**). While the under 50 population was not our primary interest, it is interesting to note that our findings conform to the only previous study in the literature [11].

The second major goal in this study was to examine whether the number of γ-H2AX foci correlated with clinical conditions that are connected with accumulation of genetic damage. In particular, we hypothesized that participants exposed to environmental stressors would likely elicit a positive correlation between γ-H2AX foci and disease state. We examined patients exposed to IR through prostate cancer treatment, patients likely to have lower O_2_ levels at night (sleep apnea) and patients with low vitamin D levels.

γ-H2AX formation after irradiation treatment has been rather extensively studied due to the preponderance of patients receiving irradiation to treat their cancer. The rise in γ-H2AX foci/cell is quick and strong. Zwicker *et al.* [14] have shown that within 10 minutes following IR for prostate cancer, the number of lesions per nuclei isolated from the patient’s circulating lymphocytes increased >8 fold. Our prostate cancer patients who were previously treated in the past with IR had a sizeable increase of 44% of γ-H2AX foci/cell over those with no known IR treatment, but these results did not achieve statistical significance (*p* = 0.23).

Sleep apnea, where repetitive obstruction of the upper airway during sleep leads to cycles of oxygen saturation/desaturation that are analogous to repetitive episodes of ischemia-reperfusion injury, is believed to result in reactive oxygen species (ROS) formation during reoxygenation [15]. One study [15] found an increase in 8-hydroxy 2-deoxyguanosine (8-OHdG; formed following the oxidation of guanine residues) in the urine of sleep apnea patients. The urinary 8-OHdG levels correlated with both the severity of the apnea, as measured by the apnea-hypopnea index, and the oxygen desaturation index. A second study [16] found that there was a higher basal level of lymphocytic DNA damage (as measured by Comet assay) from patients with sleep apnea and there was also an increased sensitivity of their lymphocytes to DNA damaging agents including hydrogen peroxide, IR and alcohol. We similarly show an increase of DNA damage in cells from sleep apnea patients (44% increase) but it did not reach statistical significance (*p* = 0.097).

Vitamin D has been known to have a protective effect against DNA damage while vitamin D deficiency is thought to be associated with impaired DNA repair capacity. Vitamin D in itself is an antioxidant and protects against sister chromatid exchange and DNA fragmentation [17]. Vitamin D has also been shown to provide partial protection against the DSBs caused by injections of diethylnitrosamine in rats [17]. In addition, when added to culture, vitamin D protected immortalized human keratinocytes and squamous cell carcinoma cells against IR, as shown by a reduction of γ-H2AX foci/cell [18]. A recent paper demonstrated that vitamin D treatment stabilized 53BP1 and promoted DNA DSBs repair via inhibition of cysteine protease Cathepsin L which lead to a decrease in cells with >5 γ-H2AX foci in lamin A-deficient (*Lmna* ^−/−^) mouse embryonic fibroblasts [20]. The results of our analysis showed a significant (*p* = 0.023) 50% increase in γ-H2AX foci/cell (5.99±0.90 vs. 4.00±0.27) (**Table 1**) in cells from patients with vitamin D deficiency (**Fig. 3A**), suggesting a protective role for vitamin D against DNA damage in a clinical setting.

We analyzed three complex diseases that had strong genetic components to their causation: cancer, cataracts and hypertension. The lack of association of γ-H2AX foci with cancer was somewhat surprising given that cancer cells have been found to have increased endogenous γ-H2AX levels compared to normal cells [21]. γ-H2AX, however, may have a tumor suppressor effect. Several studies have shown that elevated γ-H2AX in premalignant lesions have a tumor-suppressing function promoting cell cycle arrest and senescence which has also been demonstrated in knock-out mice [2]. In addition, the cancers in our study represent non-aggressive cancers such as basal cell carcinoma and prostate cancer. Studies similar to ours need to be conducted on a broad range of cancer patients, including hematological cancers and metastatic malignancies, to analyze cancer-specific associations.

We found a trend of increased γ-H2AX foci (34%, *p* = 0.099) in patients with cataracts. A previous study [22] showed an increase in 8-OHdG in leukocytes of cataract patients. Interestingly, several studies reported increased formation of cataracts under conditions of deficient DNA repair. This is particularly evident in mouse models with DNA repair deficiencies [23], [24].

Hypertensive participants had a statistically significant (*p* = 0.046) 30% increase in γ-H2AX foci/cell when compared to non-hypertensive participants (4.97±0.51 vs. 3.82±0.30) prior to adjustment for age and sex. This significance did not hold up when adjusted for age and sex (*p* = 0.13). However, after the change-point (age ≥57 years old) where γ-H2AX foci/cell stabilizes with age, hypertensive patients had a statistically significant (*p* = 0.037) 36% increase in γ-H2AX foci/cell when compared to non-hypertensive participants (5.22±0.36 vs. 3.84±0.32, **Table 1** and **Fig. 3C**) following adjustment for age and sex. The relationship between hypertension and γ-H2AX foci might begin earlier than 57 years of age as we found that analysis of patients 44 years of age and older also revealed a statistically significant (*p* = 0.020) 33% increase in γ-H2AX foci/cell in hypertension patients over non-hypertensive patients (5.22±0.48 vs. 3.92±0.30, respectively). Recent studies by two groups have shown that there is an increase in lymphocytic DNA damage (as shown by Comet assay) and a decrease in total antioxidant status (as shown by iron reduction) in hypertension patients as compared to normal controls [25], [26]. One of these groups [26] had previously showed a correlation between lymphocyte DNA damage as shown by Comet assay and concentric left ventricular hypertrophy in hypertensive patients.

The fact that six of the seven patients in our study with the highest γ-H2AX foci/cell had hypertension points toward an association of γ-H2AX formation/DSBs in the pathogenesis of hypertension. Whether there is a global increase in genotoxic stress in hypertensive patients presumably from an increase of ROS that is detectable in circulating lymphocytes, or whether the increase in circulatory pressure seen in hypertension is leading to shear stress effects on lymphocytes resulting in DNA damage (similar to the increase in damage we see with increased G forces with higher cytocentrifugation spin rates of our live cell experiment, **Figs 1C, 1D and 1E**) remains unclear. Animal and human studies show a clear association between hypertension and increased vascular oxidative stress secondary to the activation of ROS sources affecting endothelial cells including eNOS uncoupling, Nox, xanthine oxidase and the mitochondrial respiratory chain [27]. Hypertension leads to increased shear stress of vascular endothelial cells [28]. There are no reports of the effect of *in vivo* shear stress on lymphocytes though *in vitro* studies [29] have reported enzyme release from leukocyte granules and/or cell rupture in shear stress conditions. In one of the few experiments to look at the phenomenon, DNA damage has been reported in sperm cells subjected to increased centrifugal force [30]. Since cytocentrifugation is often used in the clinical setting, our finding of increased γ-H2AX foci in live cells exposed to higher G forces suggest that the lowest centrifugation speed possible should be used in preparing live samples or cells should be fixed prior to cytocentrifugation, as was done in our analysis of BLSA samples.

**Figure 5:**
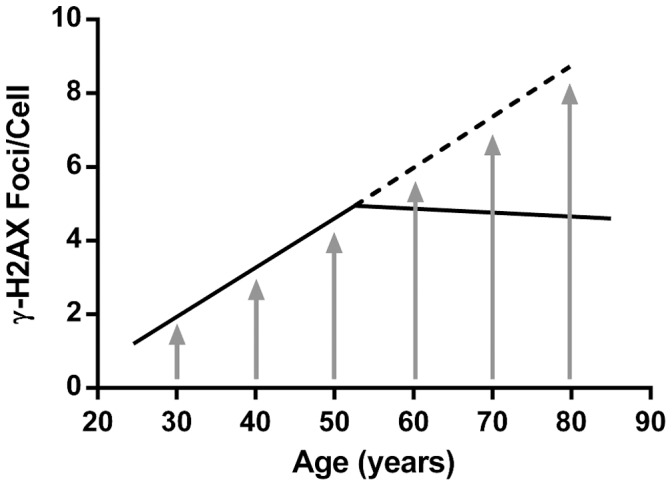
Theoretical vs. actual pattern for average number of γ-H2AX foci/cell by age. Expected increase of the average number of γ-H2AX foci/cell for each age group would theoretically mimic the prevalence of disease for that age group (shown by arrows) given that healthier patients have lower counts and morbid patients have higher counts. Solid line models actual patterns for Sedelnikova *et al.* [11] and the present study. The dashed line represents theoretical averages if a cross-section for an entire age group for a given population could be measured. Many patients, however, with morbidities such as metastatic cancer, dementia, heart disease and diabetes may not volunteer, be available or be eligible for sampling studies thus possibly skewing results toward healthier indices.

It appears from the results of our study and other studies that individuals with morbidity have higher γ-H2AX foci counts than those in a generally healthy state. Since disease prevalence in general rises with increasing age (hypertension, for example has only a 6 percent prevalence rate before age 35 but rises in a linear fashion to 78% in those 75 and older) [31] we would expect a concurrent rise in γ-H2AX foci (**Fig. 5**, upward arrows and solid line). Patients who have many comorbidities may be predicted to have increased γ-H2AX foci similar to our patient who had the most co-morbidities under study (four: sleep apnea, vitamin D deficiency, cataracts and hypertension) and who had the highest γ-H2AX foci/cell ratio (9.19 vs. 4.25 average for all participants). However, the predicted rise of γ-H2AX foci with age did not occur after the 6^th^ decade in either our study or in the study by Sedelnikova *et al.* [11] (**Fig. 5**, dotted line). Since both studies used volunteers who were likely healthier than age-matched patients in the general population, a cohort which included patients with dementia, metastatic cancer, heart disease, and diabetes may have revealed a slope of the γ-H2AX levels after age 50 with an upward trajectory.

Our survey of γ-H2AX formation over a spectrum of clinical diseases including those with environmental stressors such as vitamin D deficiency and those with a strong genetic component such as hypertension supports a role for increased DNA damage in the morbidity of age-related diseases. γ -H2AX may be a biomarker for human morbidity in age-related diseases.

## Materials and Methods

### Study Population

The present study represents analyses in an on-going population-based cohort study of normal aging and age–related diseases. The BLSA is an open cohort study, begun in 1958, of predominantly Caucasian middle to upper class individuals mostly from the Baltimore, MD and Washington, DC area who participate in follow-up assessment visits approximately every two years [32]. The study population included initially only men, and women were added in 1978. Disease data were obtained from the patient’s last visit. We analyzed data from 40 BLSA participants (26 men and 14 women, age range 35–83 years) who underwent leukapheresis from 2008 to 2011.

### Ethics Statement

The BLSA functions under the approval of the Medstar Research Institute Institutional Review Board. Participants provided written informed consent for all analyses included in this report.

### Leukapheresis

Candidates for leukapheresis (18 years or older) were screened for the following exclusion criteria: significant abnormalities found on the health history questionnaire or in the results of blood tests; pregnant or nursing mother; inadequate venous access for the cell collection procedure; severe infection in the past two months; on any medication that can alter white blood cell function; a medical or mental health finding that showed a participant could not go through leukapheresis; less than six weeks since participation in another research study which was felt by the Principal Investigator to be incompatible with the study; and/or test results were positive for viral infections.

### Ficoll Separation and Fixation

Our methods for preparation and imaging of slides are similar to the methods described by Sedelnikova and colleagues [33] Leukapheresis material was diluted using 1∶1 volume ratio of 1X phosphate buffered saline (PBS). Approximately 5 mL of diluted leukapheresis solution was carefully added as a layer on top of 4 mL Lymphocyte Separation Media (Biowhittaker) in a 15 mL falcon tube. Tubes were spun at 2000 rpm for 24 min for complete separation (slow brake speed). Plasma was removed from the top layer leaving ½ inch and the PBMC layer was collected using a disposable Pasteur pipette. Approximately 3 mL (2X) of PBMCs were treated with 5 mL of ACK lysing buffer (Biowhittaker) for 10 min at room temperature. Forty - 45 mL of PBS plus bovine serum albumin (BSA, 2% solution) was then added and the solution was centrifuged at 1600 rpm for 10 min. Supernatant was removed and 50 mL PBS plus BSA was added to cells for a second wash at 1600 rpm for 10 min. Supernatant was removed and 4% formaldehyde was added to 8 million cells for 10 min at room temperature for proper fixation. Cells were then spun at 1600 rpm for an additional 10 min. Formaldehyde was removed and 3 mL of PBS plus BSA solution was added.

### Cytocentrifugation

Lymphocytes (∼270,000cells/100 uL per slide) were spun onto slides using the Cytospin3 (Shandon) at 200 rpm for 3 mins at low acceleration.

### Staining

Cells were permeabilized using 0.5% Triton-X for 10 mins at 4°C, washed once with 1X PBS and blocked using 10% goat serum at 4°C overnight. Slides were incubated with a mouse anti- γ-H2AX primary antibody (Upstate, 1∶1000) for 1 hour at 37°C, washed 3 times with 1X PBS (10 min/wash) and then incubated with an Alexa Fluor 568-conjugated goat anti-mouse secondary antibody (Invitrogen, 1∶1000) for 30 mins at 37°C. The slides were washed 3 times with 1X PBS (10 min/wash). Nuclei were counterstained with DAPI using Prolong Gold.

### Imaging

Cells were visualized by using a Zeiss Axiovert 200 M fluorescence microscope (Zeiss, Thornwood NY). A minimum of two images (∼15 to 50 cells per image) were taken randomly of each of four slides at 100X magnification. The exposure times for each channel were kept constant; 500 ms for brightfield, 250–450 ms for CY3 and 30–75 ms for DAPI. Zeiss files were converted to TIFF files for analysis.

### Quantitation of γ-H2AX Foci

Enumerating foci per nucleus is considered the most rigorous approach compared to two other methodologies (measuring total nuclear fluorescence and scoring foci positivity) for measuring biomarkers of the DNA damage response [12]. We wished to follow a pre-set algorithm to remove operator bias from image analysis of the TIFF files. In general, processing images involves application of mathematical functions to an image to enhance brightness or contrast, reduce noise or to enhance or suppress features [12]. All images were identically processed using auto features of Adobe Photoshop (version 7.0) which has been used in automation of image analysis for immunohistochemical staining [34]. Only foci within the DAPI stained nucleus were counted. Negative controls consisting of slides stained with secondary-antibody only were devoid of foci. Deformed or fragmented nuclei, presumably corresponding to apoptotic cells, were excluded from the analysis. Patient’s age, gender and disease profile were blinded to the counter.

We had a higher number of γ-H2AX foci/cell than what other studies [11] have shown for samples derived from peripheral blood through venous aspiration (4.25 vs. <1.0 average γ-H2AX foci/cell). We believe the difference can mostly be explained by counting methodology. γ-H2AX foci may be extremely small, occupying thousands of bps or quite large, occupying up to 30 Mbps. In addition, the foci may occupy several planes along the vertical axis of the nuclei making counting difficult. We found that not overlaying the Cy3 (γ-H2AX staining) channel with the DAPI (nuclear staining) channel and counting the Cy3 channel alone improved contrast and resolution of γ-H2AX foci, thus allowing more foci to be counted (**Figs 1A and 1B**). This increased the reliability of differences between cells and consequently patients, because almost every cell would contain countable foci as opposed to only a few cells containing foci as seen in other studies.

### Choice of Diseases to Study

Cancer irradiation treatment [14], sleep apnea (reduced oxygenation) [15], [16] and vitamin D deficiency [17]–[19] are all known to be associated with genome instability and/or oxidative stress/DNA damage and were viewed as possible ‘positive controls.’ Benign prostatic hyperplasia, which is not known to be associated with genome instability or oxidative stress, was also examined as a possible ‘negative control,’ a disease that one would not expect to see increased γ-H2AX foci.

We investigated complex diseases with a heavy genetic component, specifically cancer, cataract formation, and hypertension. These conditions are often manifested in premature aging syndromes and in individuals over age 65.

Several other diseases including atherosclerotic vascular disease, diabetes and dementia, commonly found in premature aging syndromes and common in the elderly, were excluded from our study because of a paucity of patients with these ailments in our cohort likely due to stringent eligibility criteria and selection tendency of these patients not to volunteer.

### Use of Live Cells to Test the Effects of Increasing Centrifugal Force on γ-H2AX Foci Formation

Transformed human lymphocytes (GM16118 [34]) were prepared in an identical fashion as the fixed lymphocytes from BLSA participants described above, except no formaldehyde was used in the preparation. RPM speeds used were 200, 350 and 500.

### Statistical Analysis

Linear regression was used to estimate the difference of the γ-H2AX foci frequency between the groups with and without conditions, e.g., cancer versus non-cancer, after adjusting for age and gender. A p-value <0.05 was considered statistically significant. To examine the age-dependent pattern for average number of γ-H2AX foci/cell, a piecewise linear regression (change-point) model [13] was fitted with covariates age and max(0, age – change point). The change-point model assumes that the number of γ-H2AX foci/cell follows a different linear regression before and after the change point. The location of the change point was estimated as the value that gives the best overall model fit. The standard errors and the p-values of the regression parameters were calculated by assuming that the estimated change point is fixed. Because the uncertainty of the estimated change-point is not incorporated into the estimation of the parameters, the standard errors of the parameters are under estimated. Therefore, the p-values of the model parameters tend to be biased downward [35]. Statistical analyses were performed using R statistical software Version 2.15.1 [36].
